# E-Cigarette Use Among persons With Diagnosed HIV in the U.S.

**DOI:** 10.1016/j.focus.2022.100056

**Published:** 2023-03

**Authors:** Stacy L. Thorne, Ralph S. Caraballo, Yunfeng Tie, Norma S. Harris, R. Luke Shouse, John T. Brooks

**Affiliations:** 1 Division of HIV/AIDS Prevention, National Center for HIV, Viral Hepatitis, STD, and TB Prevention, Centers for Disease Control and Prevention, Atlanta, Georgia;; 2 Office on Smoking and Health, National Center for Chronic Disease Prevention and Health Promotion, Centers for Disease Control and Prevention, Atlanta, Georgia

## Abstract

**Introduction:**

E-cigarettes emerged in the U.S. market in the late 2000s. In 2017, E-cigarette use among U.S. adults was 2.8%, with higher use among some population groups. Limited studies have assessed E-cigarette use among persons with diagnosed HIV. The purpose of this study is to describe the national prevalence estimates of E-cigarette use among persons with diagnosed HIV by selected sociodemographic, behavioral, and clinical characteristics.

**Methods:**

Data were collected between June 2018 and May 2019 as part of the Medical Monitoring Project, an annual cross-sectional survey that produces nationally representative estimates of behavioral and clinical characteristics of persons with diagnosed HIV in the U.S. Statistically significant differences (*p*<0.05) were determined using chi-square tests. Data were analyzed in 2021.

**Results:**

Among persons with diagnosed HIV, 5.9% reported currently using E-cigarettes, 27.1% had ever used them but were not using them currently, and 72.9% had never used them. Current use of E-cigarettes was highest among persons with diagnosed HIV who currently smoke conventional cigarettes (11.1%), those with major depression (10.8%), those aged 25–34 years (10.5%), those who reported injectable and noninjectable drug use in the past 12 months (9.7%), those diagnosed <5 years ago (9.5%), those who self-reported sexual orientation as other (9.2%), and non-Hispanic White people (8.4%).

**Conclusions:**

Overall, findings suggest that a greater proportion of persons with diagnosed HIV used E-cigarettes than the overall U.S. adult population and that higher rates were observed among certain subgroups, including those who currently smoke cigarettes. E-cigarette use among persons with diagnosed HIV warrants continued attention because of its potential impact on HIV-related morbidity and mortality.

## INTRODUCTION

In the late 2000s, E-cigarettes emerged in the U.S. market and were initially advertised as a cessation aid to those who smoke cigarettes.^1^ These battery-powered devices deliver nicotine, flavoring, and other additives through an inhaled aerosol.^1^ Since the emergence of E-cigarettes in the U.S. and world markets, minimal information exists about potential long-term health effects. However, there are studies on the short-term effects of E-cigarettes.^2^ Studies have linked E-cigarette use to adverse cardiovascular and respiratory outcomes.^3,4^ Notably, ingredients in E-cigarettes vary, including various nicotine concentrations, carcinogens, and toxic substances found in tobacco cigarettes.^1^ Although there are some common carcinogens in E-cigarettes and cigarettes, overall, E-cigarettes appear to contain fewer amounts of carcinogens^5–8^ and may benefit those trying to quit smoking, if used as a complete substitute for combustible tobacco products.^9^

Over time, the use of E-cigarettes has increased among various population groups, especially youth (aged 13–18 years), young adults (aged 18–24 years), and those who currently smoke cigarettes.^10,11^ During 2018–2019, E-cigarette use among U.S. adults was 2.3% and was higher among some population groups.^12^ About 39% of adults who currently use E-cigarettes also currently smoke cigarettes (dual users),^12,13^ which may lead to increased nicotine dependency and higher risks of tobacco-related morbidity and mortality.^3,14^

Since 2009, cigarette smoking among persons with diagnosed HIV (PWH) has decreased; however, usage remains significantly higher, and PWH are less likely to quit than the general U.S. population (33.6% vs.16.8%).^15^ Risks of HIV and non-HIV–related morbidity and mortality due to cigarette smoking are higher for PWH, including those taking antiretroviral medications (ARTs).^16^ Even though E-cigarettes can serve as a bridge to tobacco cessation among persons who currently smoke cigarettes, the health effects, such as lung diseases, associated with their use may pose similar health risks among PWH, similar to that of the general population.^3,6,14^ At present, estimates of E-cigarette use among PWH are scarce. The purpose of this study is to describe the national estimates of E-cigarette use among PWH by selected sociodemographic, behavioral, and clinical characteristics.

## METHODS

### Study Population

Data were obtained from the Medical Monitoring Project (MMP), an annual cross-sectional survey designed to produce nationally representative estimates of behavioral and clinical characteristics of U.S. adults diagnosed with HIV. Briefly, the 2018 and 2019 MMP data cycles used a 2-stage sampling method that has been described elsewhere.^17^ MMP data collection has been determined to be nonresearch.^18^ Participating states or territories obtained local IRB approval, when necessary, on the basis of local requirements to collect data and obtained informed consent from all participants. Data were weighted on the basis of known probabilities of selection at state/territory and person levels and to adjust for person-level nonresponse and were poststratified to National HIV Surveillance System population totals.^17^ Data were combined from participant interviews and medical record abstraction collected during MMP’s 2018 and 2019 data cycles (*n*=8,150) to assess the prevalence of E-cigarette use among PWH.

### Measures

*Persons who currently use E-cigarettes* were defined as persons who reported having used ≥1 E-cigarettes in their lifetime and in the past 30 days. *Persons who have ever tried E-cigarettes* were defined as individuals who had used ≥1 E-cigarettes in their life time but not in the past 30 days. *Persons who have never tried E-cigarettes* were defined as individuals who had never used an E-cigarette.

Self-reported information on sociodemographic and behavioral characteristics from participants was included. Sociodemographic variables included sex, sexual orientation, race/ethnicity, age, educational attainment, health insurance or other coverage for medical expenses, and annual household income. Household income and the number of household dependents were used to determine participants’ poverty level on the basis of guidelines and thresholds published by the HHS, Census Bureau for 2017–2019.^19^ Health insurance was categorized on the basis of participant’s self-report regarding the type of coverage during the 12 months before the interview. Behavioral characteristic variables included the use of cigarettes, alcohol, and other substances as well as diagnosis of depression. Utilizing an established definition for smoking,^15^ persons who currently smoke were individuals who smoked at least 100 cigarettes in their lifetime and currently smoked daily, weekly, monthly, or less than monthly. Persons who formerly smoked were individuals who reported that they had smoked ≥100 cigarettes in their lifetime and currently did not smoke, whereas persons who never smoke were individuals who reported that they had smoked 0 to <100 cigarettes in their lifetime. *Any alcohol use* was defined as having consumed ≥1 alcoholic beverage during the 12 months before the interview. *Any drug use* was defined as having used injected or noninjected drugs during the past 12 months. Drugs assessed include both illicit and prescription drugs. Prescription drugs could have been nonprescribed or prescribed but taken more than directed. As described elsewhere, self-reported responses to the Patient Health Questionnaire depression scale were used to determine whether participants had major, other, or no depression.^20^

Also included were HIV clinical variables abstracted from participants’ medical records. These variables included time since HIV diagnosis, HIV disease stage at diagnosis, prescribed ART, and recent or sustained viral suppression. *Recent viral suppression* was defined as the most recent viral load measurement in the past 12 months <200 copies/mL. *Sustained viral suppression* was defined as having viral load measurements <200 copies/mL on all viral load measurements in the past 12 months.

### Statistical Analysis

Weighted percentages and associated 95% CIs were computed. Statistical estimations were suppressed if the sample size was <30 or the relative coefficient of variation was >0.30. Statistically significant differences (*p*<0.05) were determined using chi-square tests. All analyses accounted for complex sample design and unequal selection probabilities and were conducted using SAS, Version 9.4. Data were analyzed in 2021.

## RESULTS

Descriptive data for the 8,150 MMP participants included in this analysis are shown in Table 1. During 2018–2019, 74.7% of the study population was male (CI=73.0, 76.5), 46.3% were heterosexual (CI=43.6, 49.0), and 41.4% were Black Americans (CI=34.8, 48.0). The median age was 50 years, and 54.3% had public insurance (CI=51.5, 57.0) other than Ryan White HIV/AIDS or AIDS Drug Assistance Coverage. In addition, 67.6% had HIV for >10 years (CI=66.5, 68.7), 93.7% were currently taking ART (CI=92.8, 94.6), 67.7% were virally suppressed at the time of their most recent viral load test (CI=64.7, 70.6), and 61.6% had sustained viral suppression (CI=58.9, 64.2). At least 32% of the study population were persons who currently smoke cigarettes (CI=30.0, 33.9), 62.1% used alcohol in the last 12 months (CI=59.9, 64.2), and 33.0% used injection or noninjection drugs in the last 12 months (CI=30.9, 35.0). In the study population, 5.9% currently used E-cigarettes (CI=5.2, 6.5), 27.1% ever used (but not currently) an E-cigarette (CI=24.6, 29.7), and 72.9% had never used E-cigarettes (CI=70.3, 75.4).

Current E-cigarette use among PWH was about 2 times higher among males (6.7%, CI=5.9, 7.5) than among females (3.3%, CI=2.3, 4.2). Current E-cigarette use among PWH was also about 2 times higher among those who reported being homosexual or gay (8.0%, CI=6.8, 9.1) or bisexual (6.4%, CI=4.4, 8.4) than among those who reported being heterosexuals (3.7%, CI=2.9,4.5) (Table 2). Current E-cigarette use was about 2 times higher among White Americans and others than among Black Americans (8.4%, CI=7.3, 9.6, 7.3% and CI=5.0, 9.6 vs 3.9%, CI=3.0, 4.7, respectively). Estimates of current E-cigarette use decreased with age; among the age groups with sufficient sample size for robust statistical estimation, use was highest among those aged 25–34 years (10.5%, CI=8.5, 12.5). Estimate of current E-cigarette use increased with education attainment; use was highest among those with more than a high school diploma (6.5%, CI=5.7, 7.4, *p*<0.05). Current E-cigarette use was also highest among participants whose HIV diagnosis was <5 years ago (9.5%, CI=7.6, 11.4) compared with among those who were diagnosed >10 years ago (4.7%, CI=4.0, 5.4).

Among the HIV clinical characteristics, current E-cigarette use was almost 2 times higher among PWH who were not in HIV disease Stage 3 than among those who were (7.1%, CI=6.1, 8.0 vs. 4.9%, CI=4.1, 5.7). Current E-cigarette use was also high among PWH who did not have sustained viral suppression (6.7%, CI=5.6, 7.8, *p*<0.05).

Current E-cigarette use was about 5 times higher among those who currently smoke cigarettes (11.1%, CI=9.7, 12.4) and 2 times higher among those who formerly smoked cigarettes (6.5%, CI=5.1, 7.9) than among those who never smoked cigarettes (2.0%, CI=1.4, 2.7). Current E-cigarette use was higher among people who used substances than among people who did not. Among persons who had used any alcohol or who used injectable and noninjectable drugs in the past 12 months, E-cigarette use was 7.2% (CI=6.3, 8.0) and 9.7% (CI=8.5, 11.0), respectively. Current use of E-cigarettes was higher among PWH who had major depression (10.8%, CI=8.1, 13.6) and other forms of depression (6.1%, CI=3.7, 8.6) than among PWH who did not have depression (5.3%, CI=4.6, 6.0).

Demographic characteristic estimates for persons who have ever tried E-cigarettes mimicked estimates for persons who are currently using E-cigarettes. These estimates can be found in Table 2. Ever use of E-cigarettes was higher among PWH whose diagnosis was not at disease Stage 3 (31.8%, CI=28.8, 34.7) than among those who were (23.4%, CI=23.8, 26.0). Ever use of E-cigarettes among PWH who were not prescribed (30.7%, CI=27.0, 34.4) or not currently taking ART (34.3%, CI=28.8,39.8) was higher than among those who were prescribed (26.3%, CI=23.7, 28.9, *p*<0.05) or currently taking ART (26.6%, CI=24.1, 29.2, *p*<0.05). Ever use of E-cigarettes was higher among those who did not have sustained viral suppression (31.4%, CI=27.8, 35.0) than among those who had sustained viral suppression (24.5%,CI=22.2, 26.7). Ever use was higher among persons who had not achieved viral suppression (31.0%, CI=27.2, 34.9) than among those who had (25.3%, CI=23.1, 27.5) (Table 2).

Ever E-cigarette use was higher among persons who currently smoke cigarettes (51.0%, CI=47.2, 54.8) and persons who formerly smoked cigarettes (30.2%, CI=27.4, 32.9) than among persons who never smoked cigarettes (9.5%, CI=8.1, 11.0). Ever E-cigarette use was higher among people who used substances than among people who did not. Among persons who had used any alcohol or who used injectable and noninjectable drugs in the past 12 months, E-cigarette use was 31.7% (CI=29.2, 34.1) and 44.8% (CI=42.0, 47.6), respectively. Ever use of E-cigarettes was higher among those who had major depression (41.9%, CI=37.5, 46.4) than among those who had no depression (25.0%, CI=22.4, 27.6).

## DISCUSSION

To our knowledge, these are the first nationally representative prevalence estimates of E-cigarette use among U.S. PWH. These findings suggest that current and ever use of E-cigarettes among PWH is higher than among the general U.S. population.^12^ Findings showed that nearly 1 in 4 PWH had tried using E-cigarettes and that 1 in 20 PWH were current users. Even though E-cigarettes have only been in the U.S. market for about 10 years, evidence is emerging that E-cigarette use may cause deleterious health effects, especially for young users.^3,4^

Although this study group was an older cohort, with a median age of 50 years, only 2% were between the ages of 18 years and 24 years; we also found that current E-cigarette use varied among subgroups of PWH. Specifically, current and ever usage was higher among PWH who self-identified as bisexual, homosexual, or gay; males; non-Hispanic white people or others; those aged 25–34 years; those who had more than a high-school diploma; those who used any alcohol or drugs in the past 12 months; and those who have not sustained viral suppression.

Even though E-cigarettes were originally marketed as effective cessation aids to persons who smoke conventional cigarettes, they contain nicotine, the main ingredient, and other toxic ingredients also found in conventional cigarettes.^1^ While the emissions from E-cigarettes generally contain lower levels of harmful ingredients than the smoke from regular cigarettes, they are not necessarily safer.^21^ Research shows that dual use of E-cigarettes and conventional cigarettes increases nicotine exposure and intake, which may prolong tobacco substance use disorder and negate cessation efforts.^6,14^ The finding that approximately 11% of PWH who currently smoke conventional cigarettes had also tried E-cigarettes is consistent with general population studies regarding the dual use of E-cigarettes and conventional cigarettes.^12,13^ It is noteworthy that persons with HIV who smoke make fewer quit attempts and have lower rates of smoking cessation success than the general population.^15^ Similar to that of the general population, several behavioral risk factors such as alcohol, substance use, and mental health issues have been identified as barriers to successful smoking cessation among PWH.^22^ These barriers combined with perceptions that E-cigarettes are effective cessation aids may partially explain the higher prevalence of E-cigarette use among persons with HIV who currently smoke than among the general population. Despite the fact that E-cigarettes are not Food and Drug Administration approved for smoking cessation coupled with the uncertainty of long-term health impacts, PWH are interested in their use.^23^ E-cigarettes may have the potential to benefit non-pregnant adults who smoke conventional cigarettes if used as a complete substitute for regular cigarettes and other smoked tobacco products.^21^ In order for adults who smoke conventional cigarettes to achieve any meaningful health benefits from e-cigarettes, they must fully switch to E-cigarettes and completely stop smoking conventional cigarettes and other tobacco products.^21^ Even though less harmful cessation aids exist (e.g., nicotine replacement, pharmaceutical treatment, and cessation counseling),^24^ there is literature to suggest that PWH may use them as a bridge to tobacco cessation or a safer substitute for combustible tobacco products.^23^

Over the past 30 years, achievements in the HIV epidemic resulting in PWH living longer and healthier lives have occurred.^25^ Considering amplified health effects caused by the use of conventional cigarettes for PWH compared with that for persons in the general population,^16,24^ E-cigarette use among PWH merits close attention. To avoid a rapid increase in E-cigarette use among PWH and to sustain PWH living longer and healthier lives, monitoring efforts for E-cigarette use among PWH and interventions to deter tobacco use for PWH should continue.

### Limitations

This study has limitations. First, the analysis is limited to persons diagnosed with HIV in the U.S.; the results do not provide E-cigarette estimates among persons with undiagnosed HIV in the U.S. Second, our estimates of E-cigarette and conventional cigarette use were based on self-report and were not biochemically validated; however, studies have shown good correlation between self-reported tobacco use behaviors and biochemical measures such as cotinine.^26^ Third, although MMP used data-weighting methods to mitigate nonresponse bias, nonresponse bias is still possible. In addition, there are differences between MMP and general population surveys (e.g., National Health Interview Survey) in the definition of current E-cigarette use. Fourth, owing to population sample size and unstable estimates, we were unable to perform a multivariable regression.^27^ For example, for current E-cigarette use by age, the estimate of the proportion of current E-cigarette use in the age group 18–24 years had a coefficient variation >0.30, so it is suppressed for reporting and cannot be modeled.^27^

## CONCLUSIONS

These findings suggest that current and ever use of E-cigarettes among PWH was at a greater proportion than among the general U.S. population,^12^ suggesting that E-cigarette use may be a potential issue for PWH if they are being used with other tobacco products and not solely used as a substitute for conventional cigarettes and other smoked tobacco products. It is unclear at this time whether health effects related to E-cigarettes are amplified in the presence of HIV infection as it is for cigarette smoking.^16^ E-cigarette use may be a preventable health threat; therefore, usage should be discouraged among adults who do not smoke conventional cigarettes. Persons interested in quitting smoking should be encouraged to first try Food and Drug Administration‒ approved smoking cessation aids, especially among PWH.

## Figures and Tables

**Table 1.** Sociodemographic and HIV Clinical Characteristics of Adults With Diagnosed HIV, MMP, 2018–2019

Demographics	*n* ^a^	%^b^ (95% CI^c^)
Sex		
Male	5,888	74.7 (73.0, 76.5)
Female	2,090	23.3 (21.6, 25.1)
Transgender^d^	165	2.0 (1.6, 2.3)
Sexual orientation		
Heterosexual or straight	3,866	46.3 (43.6, 49.0)
Homosexual or gay	3,266	41.3 (38.6, 43.9)
Bisexual	715	9.3 (8.3, 10.2)
Other	238	3.1 (2.5, 3.7)
Race/ethnicity^e^		
White, non-Hispanic	2,320	29.1 (25.1, 33.2)
Black, non-Hispanic	3,459	41.4 (34.8, 48.0)
Hispanic/Latino	1,816	22.3 (16.8, 27.9)
Other	555	7.1 (5.8, 8.4)
Age at the time of interview (years)		
18–24	173	2.2 (1.7, 2.6)
25–34	1,109	14.5 (13.3, 15.6)
35–44	1,364	18.4 (17.2, 19.5)
45–54	2,265	27.6 (26.6, 28.6)
≥55	3,239	37.5 (36.0, 38.9)
Education		
Less than HS, no diploma	1,403	16.6 (15.4, 17.8)
HS diploma or GED	2,191	26.9 (25.6, 28.2)
More than HS	4,533	56.5 (54.6, 58.4)
Combined yearly household income ($)^d^		
0–19,999	3,965	52.0 (50.1, 53.9)
20,000–39,999	1,625	22.7 (21.4, 24.0)
40,000–74,999	1,035	14.6 (13.6, 15.6)
≥75,000	822	10.7 (9.2, 12.2)
Poverty guidelines^d^		
Above poverty level	4,200	57.8 (55.6, 59.9)
At or below poverty level	3,244	42.2 (40.1, 44.4)
Time since HIV diagnosis (yr)		
<5	1,132	14.5 (13.5, 15.5)
5–9	1,416	17.9 (16.9, 19.0)
≥10	5,594	67.6 (66.5, 68.7)
Health Insurance or coverage type^d^		
Private insurance	2,771	34.1 (32.0, 36.2)
Public insurance (excluding RW/ADAP only)	4,507	54.3 (51.2, 57.4)
RW/ADAP only or no insurance coverage	769	11.2 (9.1, 13.3)
Unspecified insurance	46	0.5 (0.2, 0.7)
HIV clinical characteristics^d^		
HIV disease Stage 3 (AIDS)	4,734	55.8 (54.3, 57.2)
Prescribed ART	7,032	81.9 (80.6, 83.2)
Currently taking ART	7,758	93.7 (92.8, 94.6)
Viral suppression		
Sustained viral suppression	5,409	61.6 (58.9, 64.2)
Recent viral suppression	5,974	67.7 (64.7, 70.6)
Had at least one VL (past 12 months)	6,603	75.3 (71.9, 78.7)
Geometric mean CD4 count ≥200	6,037	92.4 (91.6, 93.1)
Behavioral characteristics		
Cigarette use^f^		
Never	3,751	47.0 (44.6, 49.4)
Former	1,756	21.0 (19.5, 22.6)
Current	2,563	32.0 (30.0, 33.9)
E-cigarette use^g^		
Never	5,982	72.9 (70.3, 75.4)
Ever	2,105	27.1 (24.6, 29.7)
Current	448	5.9 (5.2, 6.5)
Any alcohol use (past 12 months)^d^		
No alcohol use	3,091	37.9 (35.8, 40.1)
Alcohol use	4,991	62.1 (59.9, 64.2)
Any drug use (past 12 months)^h^		
No injection or noninjection drug use	5,395	67.0 (65.0, 69.1)
Injection or noninjection drug use	2,659	33.0 (30.9, 35.0)
Depression^d^		
No depression	6,664	83.2 (82.0, 84.3)
Other depression	605	7.5 (6.8, 8.1)
Major depression	742	9.4 (8.5, 10.3)
Total	8,150	

a Numbers are unweighted. Numbers might not add to total because of missing data.

b Percentages are weighted column percentages. Percentages might not sum to 100 because of rounding.

c CIs incorporate weighted percentages.

d Variable definition has been described in detail in the study Centers for Disease Control and Prevention. Behavioral and Clinical Characteristics of Persons with Diagnosed HIV Infection: Medical Monitoring Project, United States 2016 Cycle (June 2016 – May 2017). In: HIV Surveillance Special Report 21; Revised edition. https://www.cdc.gov/hiv/library/reports/hiv-surveillance.html. Published June 2019.

e Non-Hispanic White: participants who self-identify as non-Hispanic and White only. Non-Hispanic Black: participants who self-identify as non-Hispanic and Black/African American only¡Hispanic participants who self-identify as Hispanic, even if other race/ethnicity categories were selected. Other participants include those who selected Asian, Native Hawaiian/other Pacific Islander, American Indian/Alaska Native, or multiple race/ethnicity categories.

f Never smoker: respondents who said that they have not smoked at least 100 cigarettes in their entire life. Current smokers: respondents who said that they have smoked at least 100 cigarettes in their entire life and who now smoke daily, weekly, monthly, and less than monthly. Former smoker: respondents who said that they have smoked at least 100 cigarettes in their entire life and who now never smoke.

g *E-cigarette ever use* was defined as respondents who said that they have used an E-cigarette even just 1 time in their entire life. *Current E-cigarette use* was defined as respondents who said that they have used an E-cigarette even just 1 time in their entire life and have used E-cigarettes during the past 30 days.

h Includes all drugs that were injected and not injected (i.e., administered by any route other than injection), including legal drugs that were not used for medical purposes.

ART, antiretroviral therapy; HS, high school; MMP, Medical Monitoring Project; RW/ADAP, Ryan White HIV/AIDS or AIDS Drug Assistance Coverage; VL, viral load.

**Table 2.** Sociodemographic and HIV Clinical Characteristics Among E-cigarette Adult Users With Diagnosed HIV, MMP 2018–2019

Demographics	Current E-cigarette use^a^			Ever E-cigarette use^a^			Never E-cigarette use^a^		
	*n* ^b^	%^c^ (95% CI^d^)	*p*-Value^e^	*n* ^b^	%^c^ (95% CI^d^)	*p*-Value^e^	*n* ^b^	%^c^ (95% CI^d^)	*p*-Value^e^
Sex			**<0.0001**			**<0.0001**			**<0.0001**
Male	367	6.7 (5.9, 7.5)		1,639	29.1 (26.7, 31.5)		4,201	70.9 (68.5, 73.3)	
Female	69	3.3 (2.3, 4.2)		411	20.5 (16.8, 24.1)		1,666	79.5 (75.9, 83.2)	
Transgender^f^	12	NA		52	31.0 (22.1, 39.9)		111	69.0 (60.1, 77.9)	
Sexual orientation			**0.001**			**<0.0001**			**<0.0001**
Heterosexual or straight	138	3.7 (2.9, 4.5)		738	19.9 (16.8, 23.0)		3,110	80.1 (77.0, 83.2)	
Homosexual or gay	246	8.0 (6.8, 9.1)		1,049	33.1 (30.8, 35.4)		2,205	66.9 (64.6, 69.2)	
Bisexual	42	6.4 (4.4, 8.4)		222	32.6 (27.1, 38.0)		485	67.4 (62.0, 72.9)	
Other	20	9.2 (4.2, 14.2)		89	41.0 (31.6, 50.5)		147	59.0 (49.5, 68.4)	
Race/ethnicity^g^			**<0.0001**			**<0.0001**			**<0.0001**
White, non-Hispanic	193	8.4 (7.3, 9.6)		829	37.4 (34.3, 40.4)		1,477	62.6 (59.6, 65.7)	
Black, non-Hispanic	117	3.9 (3.0, 4.7)		698	21.7 (19.3, 24.0)		2,732	78.3 (76.0, 80.7)	
Hispanic/Latino	96	5.8 (4.3, 7.2)		384	21.4 (17.8, 25.0)		1,416	78.6 (75.0, 82.2)	
Other	42	7.3 (5.0, 9.6)		194	34.7 (29.3, 40.1)		357	65.3 (59.9, 70.7)	
Age at the time of interview (years)			**<0.0001**			**<0.0001**			**<0.0001**
18–24	16	NA		56	34.2 (25.9, 42.6)		116	65.8 (57.4, 74.1)	
25–34	109	10.5 (8.5, 12.5)		467	44.7 (40.0, 49.4)		632	55.3 (50.6, 60.0)	
35–44	104	8.0 (6.3, 9.7)		430	32.2 (29.0, 35.5)		921	67.8 (64.5, 71.0)	
45–54	120	5.1 (4.0, 6.2)		592	26.9 (23.6, 30.2)		1,658	73.1 (69.8, 76.4)	
≥55	99	3.3 (2.5, 4.1)		560	17.6 (15.0, 20.2)		2,655	82.4 (79.8, 85.0)	
Education			**0.006**			**<0.001**			**<0.001**
Less than HS, no diploma	50	3.7 (2.5, 4.8)		293	21.2 (17.8, 24.6)		1,102	78.8 (75.4, 82.2)	
HS diploma or GED	126	5.8 (4.5, 7.1)		564	27.0 (22.4, 31.5)		1,612	73.0 (68.5, 77.6)	
More than HS	272	6.5 (5.7, 7.4)		1,248	29.0 (26.8, 31.1)		3,264	71.0 (68.9, 73.2)	
Combined yearly household income ($)^f^			**0.045**			0.728			0.728
0–19,999	204	5.3 (4.3, 6.2)		1,046	27.7 (24.1, 31.4)		2,905	72.3 (68.6, 75.9)	
20,000–39,999	95	6.8 (5.4, 8.2)		436	28.2 (25.0, 31.5)		1,177	71.8 (68.5, 75.0)	
40,000–74,999	74	7.9 (6.0, 9.9)		275	27.4 (23.4, 31.3)		758	72.6 (68.7, 76.6)	
≥75,000	52	6.3 (4.3, 8.3)		198	25.3 (21.4, 29.2)		622	74.7 (70.8, 78.6)	
Poverty guidelines^f^			**0.023**			0.629			0.629
Above poverty level	258	6.8 (5.9, 7.7)		1,114	27.8 (25.2, 30.5)		3,064	72.2 (69.5, 74.8)	
At or below poverty level	167	5.2 (4.3, 6.2)		840	27.1 (23.4, 30.7)		2,396	72.9 (69.3, 76.6)	
Time since HIV diagnosis (year)			**<0.0001**			**<0.0001**			**<0.0001**
<5	97	9.5 (7.6, 11.4)		375	35.4 (31.6, 39.2)		743	64.6 (60.8, 68.4)	
5–9	95	7.3 (5.9, 8.8)		430	31.2 (27.7, 34.8)		974	68.8 (65.2, 72.3)	
≥10	256	4.7 (4.0, 5.4)		1,298	24.3 (21.7, 26.8)		4,259	75.7 (73.2, 78.3)	
Health insurance or coverage^f^			0.050			0.421			0.421
Private insurance	172	6.9 (5.7, 8.0)		681	25.9 (23.4, 28.4)		2,076	74.1 (71.6, 76.6)	
Public insurance (excluding RW/ADAP only)	219	5.2 (4.3, 6.0)		1,195	27.7 (24.3, 31.0)		3,294	72.3 (69.0, 75.7)	
RW/ADAP Only or No insurance coverage	55	6.5 (4.6, 8.4)		212	28.5 (25.0, 32.0)		553	71.5 (68.0, 75.0)	
Unspecified insurance	2	NA		12	24.1 (12.3, 35.9)		34	75.9 (64.1, 87.7)	
HIV clinical characteristics^f^									
HIV disease Stage 3 (AIDS)			**<0.001**			**<0.0001**			**<0.0001**
No	226	7.1 (6.1, 8.0)		1,041	31.8 (28.8, 34.7)		2,354	68.2 (65.3, 71.2)	
Yes	222	4.9 (4.1, 5.7)		1,063	23.4 (20.8, 26.0)		3,628	76.6 (74.0, 79.2)	
Prescribed ART			0.900			**0.009**			**0.009**
No	59	6.0 (4.3, 7.7)		309	30.7 (27.0, 34.4)		789	69.3 (65.6, 73.0)	
Yes	389	5.8 (5.1, 6.6)		1,796	26.3 (23.7, 28.9)		5,193	73.7 (71.1, 76.3)	
Currently taking ART			0.882			**0.001**			**0.001**
No	19	5.7 (2.7, 8.6)		105	34.3 (28.8, 39.8)		221	65.7 (60.2, 71.2)	
Yes	429	5.9 (5.2, 6.6)		1,996	26.6 (24.1, 29.2)		5,741	73.4 (70.8, 75.9)	
Viral suppression									
Sustained viral suppression			**0.044**			**<0.0001**			**<0.0001**
No	175	6.7 (5.6, 7.8)		810	31.4 (27.8, 35.0)		1,895	68.6 (65.0, 72.2)	
Yes	273	5.4 (4.6, 6.1)		1,295	24.5 (22.2, 26.7)		4,087	75.5 (73.3, 77.8)	
Most recent viral suppression			0.607			**<0.0001**			**<0.0001**
No	126	6.1 (4.9, 7.3)		636	31.0 (27.2, 34.9)		1,509	69.0 (65.1, 72.8)	
Yes	322	5.7 (5.0, 6.5)		1,469	25.3 (23.1, 27.5)		4,473	74.7 (72.5, 76.9)	
Had at least 1 VL (past 12 months)			0.850			**0.013**			**0.013**
No	83	6.0 (4.6, 7.3)		437	30.4 (25.8, 35.1)		1,080	69.6 (64.9, 74.2)	
Yes	365	5.8 (5.1, 6.6)		1,664	25.9 (23.7, 28.2)		4,899	74.1 (71.8, 76.3)	
Geometric mean CD4 count ≥200			0.064			0.820			0.820
No	20	4.0 (2.4, 5.6)		125	25.9 (21.0, 30.8)		384	74.1 (69.2, 79.0)	
Yes	336	5.9 (5.0, 6.7)		1,548	26.3 (23.8, 28.8)		4,452	73.7 (71.2, 76.2)	
Behavioral characteristics									
Cigarette use^h^			**<0.0001**			**<0.0001**			**<0.0001**
Never	72	2.0 (1.4, 2.7)		347	9.5 (8.1, 11.0)		3,403	90.5 (89.0, 91.9)	
Former	104	6.5 (5.1, 7.9)		503	30.2 (27.4, 32.9)		1,253	69.8 (67.1, 72.6)	
Current	271	11.1 (9.7, 12.4)		1,251	51.0 (47.2, 54.8)		1,312	49.0 (45.2, 52.8)	
Any alcohol use (past 12 months)^f^			**<0.0001**			**<0.0001**			**<0.0001**
No alcohol use	115	3.8 (2.8, 4.7)		591	19.8 (16.7, 22.8)		2,498	80.2 (77.2, 83.3)	
Alcohol use	333	7.2 (6.3, 8.0)		1,514	31.7 (29.2, 34.1)		3,476	68.3 (65.9, 70.8)	
Any drug use (past 12 months)^i^			**<0.0001**			**<0.0001**			**<0.0001**
No injection or noninjection drug use	199	4.0 (3.3, 4.7)		946	18.4 (16.0, 20.8)		4,448	81.6 (79.2, 84.0)	
Injection or noninjection drug use	248	9.7 (8.5, 11.0)		1,148	44.8 (42.0, 47.6)		1,511	55.2 (52.4, 58.0)	
Depression^f^			**0.002**			**<0.0001**			**<0.0001**
No depression	335	5.3 (4.6, 6.0)		1,621	25.0 (22.4, 27.6)		5,041	75.0 (72.4, 77.6)	
Other depression	38	6.1 (3.7, 8.6)		183	31.7 (27.4, 36.0)		421	68.3 (64.0, 72.6)	
Major depression	71	10.8 (8.1, 13.6)		279	41.9 (37.5, 46.4)		463	58.1 (53.6, 62.5)	
Total	448	5.9 (5.2, 6.5)		2,105	27.1 (24.6, 29.7)		5,982	72.9 (70.3, 75.4)	

*Note:* Boldface indicates statistical significance (*p*<0.05).

a *E-cigarette ever use* was defined as respondents who said that they have used an E-cigarette even just 1 time in their entire life. *Current E-cigarette use* was defined as respondents who said that they have used an E-cigarette even just 1 time in their entire life and have used E-cigarettes during the past 30 days.

b Numbers are unweighted.

c Percentages are weighted row percentages.

d CIs incorporate weighted percentages.

e Statistical significance within demographic, HIV clinical, and behavior characteristics using chi-square tests.

f Variable definition has been described in detail in the study Centers for Disease Control and Prevention. Behavioral and Clinical Characteristics of Persons with Diagnosed HIV Infection: Medical Montoring Project, United States 2016 Cycle (June 2016 – May 2017). In: HIV Surveillance Special Report 21; Revised edition. https://www.cdc.gov/hiv/library/reports/hiv-surveillance.html. Published June 2019.

g Non-Hispanic White: participants who self-identify as non-Hispanic and White only. Non-Hispanic Black: participants who self-identify as non-Hispanic and Black/African American only; Hispanic participants who self-identify as Hispanic, even if other race/ethnicity categories were selected. Other participants include those who selected Asian, Native Hawaiian/other Pacific Islander, American Indian/Alaska Native, or multiple race/ethnicity categories.

h Never smoker: respondents who said that they have not smoked at least 100 cigarettes in their entire life. Current smokers: *respondents* were defined as those who said that they have smoked at least 100 cigarettes in their entire life and who now smoke daily, weekly, monthly, and less than monthly. Former smoker: respondents who said that they have smoked at least 100 cigarettes in their entire life and who now never smoke.

i Includes all drugs that were injected and not injected (i.e., administered by any route other than injection), including legal drugs that were not used for medical purposes.

NA estimates are not presented because the coefficient of variance ≥30%.

ART, antiretroviral therapy; HS, high school; MMP, Medical Monitoring Project; NA, not applicable; RW/ADAP, Ryan White HIV/AIDS or AIDS Drug Assistance Coverage; VL, viral load.
